# Experiences of quality of life the first year after stroke in Denmark and Norway. A qualitative analysis

**DOI:** 10.1080/17482631.2019.1659540

**Published:** 2019-09-24

**Authors:** Synne G. Pedersen, Audny Anke, Lena Aadal, Hanne Pallesen, Siri Moe, Cathrine Arntzen

**Affiliations:** a Department of Rehabilitation, University Hospital of North Norway, Tromsø, Norway; b Department of Health and Care Sciences, Faculty of Health Sciences, UiT, the Arctic University of Norway, Tromsø, Norway; c Department of Clinical Medicine, Faculty of Health Sciences, UiT, the Arctic University of Norway, Tromsø, Norway; d Department of Clinical Medicine, Hammel Neurorehabilitation Centre and University Research Clinic, Aarhus University, Aarhus, Denmark

**Keywords:** Quality of life, stroke, recovery, self, embodiment, fatigue, qualitative research

## Abstract

**Purpose:** This study aims to explore quality of life (QOL) during the first year of recovery after stroke in North Norway and Central Denmark. **Method:** Individual in-depth interviews with 11 stroke survivors were performed twelve months after stroke onset. An interpretative, inductive approach shaped the interview process and the processing of data. **Results:** We found that QOL reflected the individuals’ reconstruction of the embodied self, which was identified by three intertwined and negotiating processes: a familiar self, an unfamiliar self, and a recovery of self. Further, we found that reconstruction of the embodied self and QOL could be framed as an ongoing and interrelated process of “*being, doing, belonging and becoming*”. Enriching social relations, successful return to work, and continuity and presence in professional support during recovery enhanced the experience of QOL. Fatigue and sustained reduced function hindered participation in meaningful activities and influenced the perceived QOL negatively. **Conclusions:** The two countries differed in descriptions of continuity and support in the professional follow-up during the recovery process, influencing the degree of encouragement in reconstructing the embodied self. Reconstruction of the embodied self is a means of understanding stroke survivors’ QOL during the first year of recovery, supporting an individualized and tailored rehabilitation practice.

## Graphical Abstract

**Figure:**
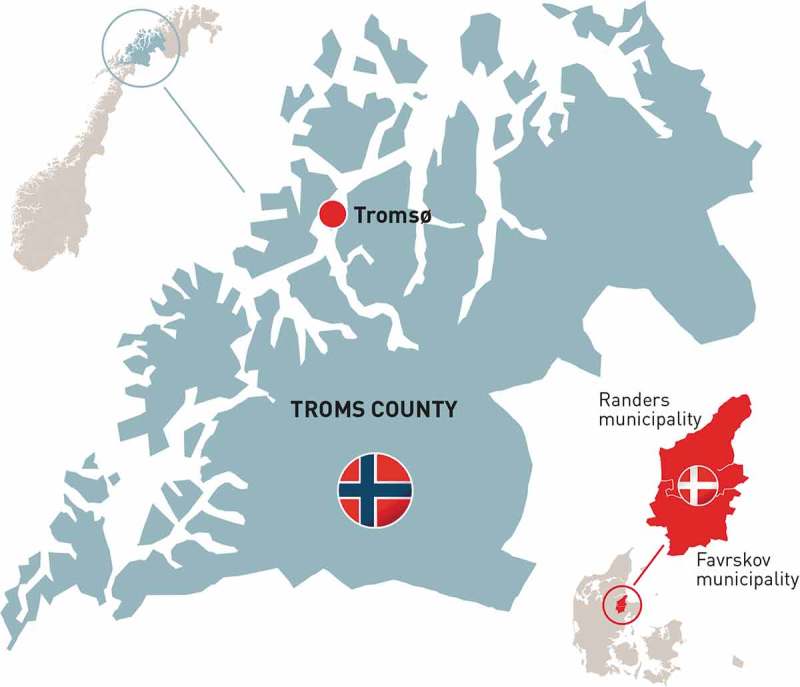

## Introduction

This study explores quality of life (QOL) one year following stroke in a region of North Norway and in the Central Denmark Region. The two Scandinavian countries are both fairly equivalent welfare societies and have similarities in life expectancy rates and cultural aspects. Geographically, different regions in North Norway are very dissimilar to Denmark, with large areas and scattered settlements. Consequently, distances to hospitals and other medical and rehabilitation centres are longer for the North Norwegian population, and some variances in health organization exist in terms of small (North Norway) and large (Denmark) units. These differences, along with local available resources, e.g., specialized professionals and individual and cultural variations in understanding rehabilitation and illness, may be important for quality of life during recovery after stroke. This study will be relevant for gaining insight into potential differences and similarities in the experienced quality of life during the first year of recovery and between two country regions after stroke.

QOL is central in stroke rehabilitation, wherein optimizing functions or adaptations to everyday life are common essential goals shared by stroke survivors and professionals (Wiklund, 2004). However, QOL is a broad and multifaceted phenomenon that may convey many meanings (Dijkers, 1999; Farquhar, 1995; Hill, Harries, & Popay, 1996; Post, 2014). Studies have implied a connection between physical function and QOL, but the results vary, with some studies implying a connection while others do not (Carod-Artal & Egido, 2009; Carod-Artal, Egido, González, & Seijas, 2000; Gunaydin, Karatepe, Kaya, & Ulutas, 2011; Samsa & Matchar, 2004; Suenkeler et al., 2002). Other studies find that QOL is a complex phenomenon that embraces more than merely physical functioning (Clarke & Black, 2005; Green & King, 2009; Kirkevold & Wyller, 1999). Although many studies have investigated QOL and its impacting factors, we have not found studies exploring QOL as experienced by stroke survivors across health-care systems and cultures. In this study, our understanding of QOL was framed through the World Health Organization’s [WHO] definition: ‘an individual’s perception of their position in life in the context of the culture and value systems in which they live and in relation to their goals, expectations, standards and concerns. It is a broad-ranging concept affected in a complex way by the person’s physical health, psychological state, personal beliefs, social relationships and their relationship to salient features of their environment’ (World Health Organization, 2018). This definition is broad and sufficiently open for empirical exploration of different aspects of the phenomenon as experienced by stroke survivors themselves.

Stroke may cause a variety of impairments, which have long-term physical, cognitive, psychological and social consequences for approximately one-third of survivors (Wolfe et al., 2011). Recovery after stroke is described as a dynamic process in which patients’ outcomes are heterogeneous and characterized by individual recovery patterns (Langhorne, Bernhardt, & Kwakkel, 2011). Several studies (Arntzen, Borg, & Hamran, 2015; Clarke & Black, 2005; Kitzmüller, Häggström, & Asplund, 2013; Meijering, Nanninga, & Lettinga, 2016; Pallesen, 2014; Sarre et al., 2014) have described different trajectories, patterns and transitions during recovery following stroke. Recovery is understood as a complex and transformative process influenced by varied and multifaceted individual and contextual interrelations. The process of recovery includes improvements in or adjustments to physical and cognitive impairments, as well as emotional and psychological post-stroke deficiencies. Therefore, recovery following stroke may be comprehended as an embodied and situated phenomenon as we experience our body in various ways depending on the context (Damasio, 1994; Merleau-Ponty, 1962). Throughout the recovery process, new meaning and purpose in one’s life that grows beyond the persisting challenges and symptoms after stroke may develop, and recovery may involve an adaptive or adjusting process, as well as achievement of the former level of functioning (Deegan, 2002). Numerous studies have investigated experiences of recovery and life following stroke (Greenwood, Mackenzie, Cloud, & Wilson, 2009; Lamb, Buchanan, Godfrey, Harrison, & Oakley, 2008; Lou, Carstensen, Jørgensen, & Nielsen, 2017; Murray, Ashworth, Forster, & Young, 2003; Peoples, Satink, & Steultjens, 2011; Salter, Hellings, Foley, & Teasell, 2008; Sarre et al., 2014; Wiles, Cott, & Gibson, 2008). These studies address different understandings, impacts, challenges and consequences of stroke from the perspectives of stroke survivors, thus, studies comparing experiences of recovery during the first year across health-care systems and cultures are scarce.

Variability in QOL (Sprigg et al., 2012), and recovery patterns between Western countries post-stroke (Ayis et al., 2015) are not fully understood; however, differences in cultural factors, health systems and available resources have been suggested (Ayis et al., 2015). Although several studies have documented different perspectives of QOL and recovery following stroke, we have not found studies investigating stroke survivors’ *experiences* of QOL in the recovery process across countries. This study aims to explore stroke survivors’ experienced QOL during the first year of recovery in North Norway and Central Denmark.

## Materials and methods

### Design

This interpretative, inductive study is part of the multicentre ‘NORDA-study’ describing and comparing stroke pathways in a region of North Norway and the Central Denmark Region. Individual semi-structured in-depth interviews (Kvale, 2007) with stroke survivors were conducted one year following stroke. Hermeneutic epistemology (Gadamer, 2004) can describe the interpretation of text and the transformation back into meaning. In this process the researchers’ interpretations involves their own interactions and experience with the world; their preconceptions are implemented in the hermeneutic phenomenological process to gain a deeper understanding of the investigated phenomenon. The dialectic movement between the text, participant and ourselves was used to seek an alternative means of interpretation rather than an illustration of a subjective point of view regarding QOL. The phenomenology of the body constituted the epistemological basis for exploring the QOL phenomenon. In this study, the lived body is understood to be situated in a dynamic physical and social life-world, implying that perception, action, awareness and emotions binds the body to the world (Damasio, 1994; Merleau-Ponty, 1962; Weiss & Haber, 1999). This overall frame of reference is useful for exploring QOL and how this phenomenon unfolds in stroke survivors’ physical, practical and social situations during the first year of recovery.

### Participants

The participants were identified stroke survivors living in comparable population-sized regions in the two countries and recruited by health personnel in hospitals. Adult participants were eligible for this study if they had a clinically confirmed diagnosis of ischaemic or haemorrhagic stroke and had physical and/or cognitive impairments requiring further rehabilitation after discharge from a stroke unit. Other inclusion criteria were that the participants had lived an independent life prior to stroke and were discharged to their homes after rehabilitation. Stroke survivors with cognitive or communicative impairments preventing them from sharing their experiences through interviews were excluded. At the time of the interviews, the age range was 35–66 years. They all lived in their own apartments or houses. Participants from Norway (n = 5) were discharged to five different municipalities, while the Danish participants (n = 6) were discharged to two municipalities (Table I).

**Table I.** Sociodemographic data

Case	Gender	Age	Country	Marital status	Work post stroke	Residents in municipality
1	Woman	<60	Denmark	Cohabiting	Retired	61.000
2	Man	≥65	Denmark	Cohabiting	Retired	61.000
3	Man	<50	Denmark	Cohabiting	Fulltime	61.000
4	Woman	<40	Denmark	Cohabiting	Work-training	48.000
5	Woman	<55	Denmark	Cohabiting	Work-training	48.000
6	Man	<55	Denmark	Cohabiting	Work-training	48.000
7	Man	<50	Norway	Single	Work-training	4.800
8	Man	<70	Norway	Single	Retired	72.000
9	Woman	<50	Norway	Single	Retired	9.500
10	Man	<45	Norway	Cohabiting	Work-training	3.500
11	Man	<60	Norway	Cohabiting	Retired	5.500

### Ethical considerations

The study was conducted according to the Helsinki Declaration regarding informed consent and confidentiality. The study was approved by the Regional Norwegian Ethical Committee, Health Region North (2013/1461) and the Danish Data Protection Agency (1-16-02-66-14). Written informed consent was obtained from all included participants prior to commencing the study.

### Data collection methods

Semi-structured interviews with stroke survivors were conducted by the authors (S.M., C.A., L.Aa., H.P.) shortly before discharge from the hospital and three and 12 months after stroke onset. The present sub-study involves the interviews conducted approximately one year following stroke. The interview guide was developed through collaboration among the authors and included topics on experiences and reflections regarding perceptions of life and the recovery process. The interviews (n = 11) ranged from 60 to 90 minutes and were audiotape-recorded with the participants’ permission. Carers were sometimes present during the interviews, and since some participants had problems with memory and/or speech, their contributions were important and helpful. All interviews were conducted in the participants’ homes or workplaces and were transcribed verbatim with identifying data removed.

### Analysis

The interdisciplinary researchers in this study have extensive experience in stroke rehabilitation. Professional competency is a key prerequisite for knowledge development, but potential may exist to make quick decisions without ruminating sufficiently on the participants’ experiences. Therefore, we carefully discussed our interpretations and challenged our preconceptions through systematic reading of the interviews and repeated discussions among the research group. Theory and research in the field were also important for implementing our pre-understanding and attaining a deeper understanding of the meaning structures in the participants’ QOL. Through systematic shifts between deductive analytic parts, the material as a whole and literature, interpretation was developed through multiple stages of understanding—the hermeneutic circle. Data were reconceptualized in an analytic text with stories, patterns and variations that shed light on the research question. The alternation between empirical data and theory reinforced the distance to the material.

**Figure 1.**
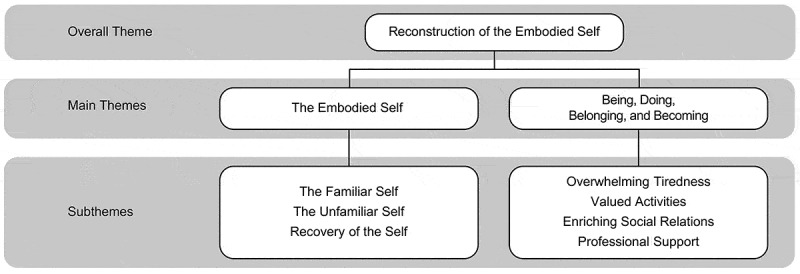
Illustration of emerging themes.

An inductive approach through systematic text condensation (STC) was used (Malterud, 2012). This approach is a pragmatic procedure inspired by Giorgi’s psychological phenomenological analysis. STC is a systematic, descriptive and explorative method for thematic cross-case analysis for qualitative data, involving analytic reduction with specified shifts between de- and re-contextualization of data (Malterud, 2012). The data were processed in NVIVO 11, where all meaning units were identified and abstracted through condensed meaning units and code-groups. The coding was discussed with last-author (C.A.) to reach a consensus regarding subgroup priority. Meaning units within the same subgroup were reduced into condensates uniting the content of the meaning units from the subgroups. A reflexive journal and a matrix for the data analysis were developed and further used as a basis for developing categories and themes. Descriptions and concepts were developed by synthesizing the contents of the condensates. The re-contextualization with interpretation and findings was systematically assessed and validated against the initial complete transcripts. Throughout the critical analysis, we asked questions: What does this mean? What is this similar to or different from? Then, similarities and differences were compared. We searched for theoretical concepts that could provide new understandings of the stories told by the participants. New interpretations emerged through literature and research on the embodied self (Gallagher, 2011; Weiss & Haber, 1999). The presented analytic text represents the most salient content and meaning that emerged from the empirical data (Figure 1, Table II). Relevant quotations are embedded in the analytic text to provide additional illustrative text elements.

**Table II.** Systematic process of de-contextualization and re-contextualization. Shortened example of data analysis. Conversation about ADL-activity and whether there are specific things that will not succeed when it comes to function

Meaning units and possible key quotes withdrawn from condensates	Code group	Condensates	Sub-category	Category	Theme
*“(…) Basically, I am back to the person I used to be”*	**Bodily changes and identity**	I think it´s going well, really. For the most part, I function the way I used to. I don´t think about activities that may not be done in in the same way anymore. If there are any, they are just unconsciously done in a different approach. There are no changes in my everyday activities, and I am doing the things that I did prior to my stroke.	Managing everyday activity Independence	Normality Back to being me	**A familiar Self**
“As long as you walk around ‘partial’, it affects you”		My life is turned upside-down, and I have to ask for help. I used to be a handy-woman, and it feels so weird not to be able to do things myself. It is a new role and I constantly dread the fact that I need to ask for help. I used to be a very social person, and now I´m afraid to go out because I can´t talk to that many people. I have to think and do things differently than I did before, and this makes me very sad—I don´t even know how to explain this. She needs to adapt another life-situation that is not her. Even though we still see glimpses of her, she is another person now. The fact that I can´t remember names disgusts me. Sometimes I start doing things automatically, and suddenly I realize that I can´t do this anymore—and this makes me furious and annoyed.	Loss Alienation Dependency	Unfamiliarity Changed perception of self	**An unfamiliar Self**
“I have accepted the situation for what it is, and I don´t get annoyed or depressed by anything now*”*		I have lost the overview in terms of following up on our practical things, and my wife is now the man in the house. Initially I thought it was terrible for myself that I could not continue doing my work, that I was good at, and the loss off my familiar co-workers. I used to take part of conversations and discussions in groups of people, but now I can´t be bothered. Earlier in the process I used to get very sad when people asked about, or responded to, the way I talked—and now this does not bother me at all. Previously I could not accept that I had to let go of my regular work. Right now, I don´t feel that I had to let go of anything.	Social re-positioning Resignation Accept	Processes of ● adjustment ● re-establishment of an adapted self Ambivalence in the process	**Recovery of Self**

## Results

### QOL and reconstruction of the embodied self

**Figure 2.**
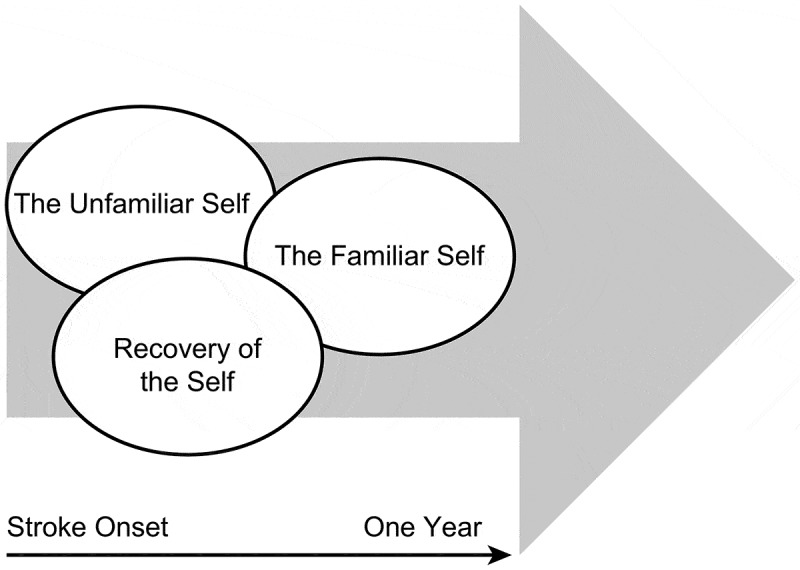
Intertwined and negotiating processes in the embodied self following stroke.

Stroke was experienced as a discontinuity in life, which essentially changed the story and conception of who they were. We found that QOL as experienced during the first year after stroke reflected a reconstruction of the embodied self. At stroke onset, unfamiliarity with the self was prominent as functional deficiencies initially represented a temporary discontinuity of self and a known way of being in the world. The experience of QOL was embedded in the *recovery of self* that gradually moved towards *a familiar self* one year following stroke (Figure 2). For a few participants, continuity of self-reconstruction was hindered due to the stroke experience and the internal battle between a familiar and *an unfamiliar self*. This intertwined and negotiating reconstruction process was essential to the person’s QOL, and will be outlined below.

### The familiar self

The narratives led to different descriptions of “I can” or “I cannot” and referred to the subject’s body and ability to move, perceive, reflect and be aware. The individuals’ possibilities in life was important for different aspects of valued activities, social participation and QOL. The participants who had restored all or most of their function reflected upon a returned normality—both generally in life and themselves as a person. A woman from Denmark provided the following statement:

*‘There are no changes in my everyday activities, and I am doing the things that I did prior to my stroke. Basically, I am back to the person I used to be.’* (case 1)

Those who no longer experienced restrictions in functioning described a continuous positive change in their self-awareness during the first year following stroke. The descriptions revealed that earlier functional disruptions, whether to the emotions or to the body, came to the foreground of awareness in different situations. Several of the participants mentioned how they used to think about *how to* do basic things in the early phase, i.e., walking, cleaning or cooking, whereas these activities were now executed pre-reflectively without thinking about or planning the corresponding actions. Self-awareness disappeared to the background as they progressed. A man from Denmark elaborates:

*‘My grocery-shopping may take a little more time … ., but no—I don’t even know if it does. I am not speculating about it anymore.’* (case 2)

The embodied knowledge in “how to do things” and the “I can” without too much struggle or thinking brought back normality and a familiar self. Descriptions from several Danish participants were portrayed as especially successful in terms of a sustained or re-established familiar self, and the same participants described a high degree of functioning and QOL. None of the Norwegian participants described the same success in regaining normality or familiarity in the embodied self.

### The unfamiliar self

Some of the participants struggled with not being who they used to be familiar with and repeatedly referred to themselves as the person before and after the stroke. Functional problems disturbed a known, familiar way of doing things and living life, thus affected their experienced QOL. In contrast to the above examples, the functional disruption and the awareness about themselves was still in the foreground in their lives. Every time they struggled in a task or in a situation, the body made itself apparent, and the awareness about what they could not achieve asserted its presence. Some participants found their functional interruption, e.g., memory loss or speech problems, repulsive because it contrasted with the image of the person that they identified themselves with—the familiar self. The breach in sense of self persisted for those who struggled with function in a profound manner and interfered with their QOL. For these participants, reconstruction of the embodied self did not progress in a positive direction after stroke. A woman from Norway claimed that her life and what she could do had changed overwhelmingly after her stroke, affecting her perception of herself. Her valued independence had turned to dependency, and her very active social life and social interactions had become challenging:

*‘My life is turned upside-down, and I have to ask for help. I used to be a handy woman, and it feels very weird not to be able to do things myself. It is a new role and I constantly struggle with the fact that I need to ask for help. I used to be a very social person, and now I am afraid to go out. I have to think and do things differently than I did before, and this makes me very sad—I do not even know how to explain this. I do not know if I will ever accept this situation. (…) I am basically dependent on others. I do not feel free—not the way I used to be.’* (case 9)

Some participants struggled to accept the breach in who they used to be in the world, and the new circumstances were parts of the self was unfamiliar because of different functional problems that ultimately changed their QOL. Participants from both countries described unfamiliarity in life and self, but only the Danish participants described receiving professional help to sustain their self-constructing process by focusing on unfamiliar aspects of self.

### Recovery of the self

Some of the participants recognized their persistent functional problems and talked about acceptance and adjustment to the new situation. The acceptance and adjustments increased the participants’ experience of QOL. Numerous participants from both countries told stories about the changes that they had been through and described the alteration from feeling sad, upset, annoyed, anxious or depressed (early in the process) to not responding in this manner at all to their present functional problems. Adjustment and acceptance gradually moved forward to a more settled, adapted embodied self.

*‘Previously, I could not accept that I had to let go of my regular work. Right now, I do not feel that I had to let go of anything.’* (case 4)

Several participants from both countries seemed to be able to re-establish an adapted self and a form of new normality through their acceptance and despite their losses in valued activities and social involvement. Although some described an ambivalence in their acceptance of certain embodied changes:

*‘I have accepted my situation, but it is sometimes hard for me to accept that I cannot accommodate the outlook that I had before.’* (case 4)

Recovery of the embodied self illustrates a process interconnected with acceptance, progress, adjustments and management in life. Despite persistent functional problems, most of the participants were able to put their experience of stroke in perspective one year later, and several participants expressed gratitude for the currently regained QOL.

### QOL and dimensions of being, doing, becoming and belonging

**Figure 3.**
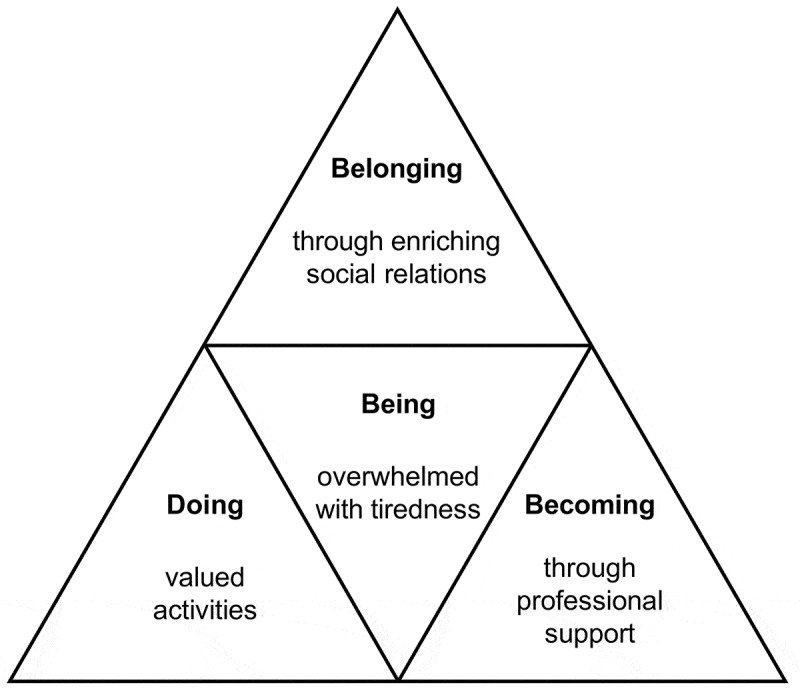
Interconnected aspects relevant for reconstruction of the embodied self.

Reconstruction of the embodied self can be understood as an ongoing and interrelated process of “being, doing, belonging and becoming” (Hitch, Pépin, & Stagnitti, 2014a, 2014b; Wilcock, 1998, 1999). These aspects conceptualize *being* as humans and may provide an understanding concerning essential human desires and possibilities significant for QOL. In this study, we found that *being* is linked to doing (action or engagement), belonging (relationships and connectedness) and becoming (a perceptual process of change and development). Thus, bodily changes or disruptions could interfere with these dimensions and affect QOL (Figure 3).

### Being overwhelmed with tiredness

An overshadowing consequence of persistent tiredness followed all the participants one-year post-stroke. Tiredness still influenced everyday life regardless of how well the participants had recovered. The individuals’ conception of their own capacity influenced their experience of QOL. They all emphasized that such tiredness represented a change in their lives that they did not experience prior to stroke.

*‘My biggest issue right now is that I need an hour to rest in the afternoons—it is a necessity to function the rest of the day and gain some energy. It* [the tiredness] *is a little bit better now, but without my hour of rest, I will not make it through the day. It is a disadvantage in my life.’* (case 2)

All of the participants talked about changed capacity or energy levels in relation to other people, work and leisure activities Almost everyone told stories of how they protected themselves from energy-consuming activities or participation throughout the day. The tiredness was portrayed as a disruptive phenomenon, influencing their overall existence—their *being*, hence influencing reconstruction of the embodied self and QOL. When tiredness precluded the participants from activities or social function, it impacted on how they saw themselves and how they assessed their own capacity in life through the “I can” or “I cannot”. None of the participants from Norway explicitly described external support to help manage the tiredness. In contrast, the Danish participants portrayed insightful knowledge of strategies for recreation to manage in everyday life throughout the recovery process, and they expressed that professional support directed them towards compensating strategies, cognitive functioning and language barriers, all of which increased their overall capacity during the day.

### Doing valued activities

Some factors threatened reconstruction of the embodied self and QOL more than others. The ability to work was particularly central to the participants’ described self-worth in both countries. Work was portrayed as self-identity by most of the participants and had a profound impact on how they saw themselves, i.e., as valuable, productive and useful individuals in society. Most of the participants had a changed or reduced work status one year after injury. While a few participants articulated satisfaction with this arrangement, others clearly missed their regular jobs and expressed uncertainty, thus hope for full-time jobs again. A man from Norway who was under organized work training elaborates:

*‘It is a place with other people, and you do not just sit at home. I think the psychological perspective of being there is very important. (…) It has been a very positive experience in numerous ways. But it is not my job—nothing of what I do is me. Although, I am being useful, I can see results in my functioning and in the job, so it is positive. Still, I would like to get back to my profession, and the goal is to get back to my job—the job that is me (…) It has a lot to do with quality of life—To work with what you really like doing.’* (case 7)

The job that he had previously performed was strongly related to his embodied familiar self. Although temporary employment with unfamiliar work tasks was not what most of the participants hoped for, they generally adjusted to the situation and were pleased to contribute something in everyday life.

The ability to drive was another valued activity important for QOL. Most of the participants in both countries spoke of enhanced life quality after they had received permission to drive again. Driving licenses represented a sense of freedom and an opportunity to pursue other valued activities. Some participants did not have permission to drive one year following stroke and explicitly described how this affected their QOL. A Norwegian man who lived in a rural area and had few options for public transportation expressed his situation as follows:

*‘If it continues like this, without permission to drive, I will bore myself to death. (…) The most important thing for me right now is to get my driver’s license back so that I can continue doing the things that I used to do.’* (case 11)

Some of the Norwegian participants described isolation without their driving licenses because they lived in sparsely inhabited areas, while others who lived more centrally described restrictions in their possibilities and the sense of freedom that driving a car may supply. Restricted permission to drive influenced a few of the participants’ work situations and the possibility to proceed in their profession as a professional driver. Hence, being able to work and drive again was important for the experience of QOL.

### Belonging through enriching social relations

Interactions and continual close relationships with family, friends, neighbours and co-workers emerged as an essential aspect for reconstruction of the embodied self and QOL. Engaging in social relationships implied incorporating others into the self and appeared important for regulating behaviour and sustainment as a person following stroke. The extent of social relationships was different in each case, but all participants highlighted the importance of having some sort of social relations. Close family was described by several of the participants as especially valued in the process of recovery. Spouses, cohabitants, children or friends had become even more important to the participants after stroke, and were natural facilitators in adjusting to everyday activity. The participants’ loved ones similarly needed to adapt to a new situation, and adjustments after stroke became a common concern that created strengthened relationships. A man from Denmark describes the value of his relationships as follows:

*‘To me, having a good life means that I as a person can do the things that I appreciate and love: go fishing, be with my family and my friends. Be with them in a way that is enriching to me—and hopefully for them too. Also, having a good life, most of all, is that we as a couple are doing well.’* (case 3)

Valuable interactions with peers were highlighted as important for QOL by most of the participants. Several of these stories were related to the acute hospitalization and rehabilitation units, and some of the participants still had strong connections with their peers. These relationships were referred to as social community networks in which members cared for each other. Observing and following the recovery processes of peers provided motivation and drive to push harder in the participants’ own training and recovery. Several explained that they felt comfortable and safe in a group of peers. In particular, those who experienced aphasia, dysarthria and cognitive problems highlighted the importance of “mutual ground” and, in a sense, “levelling” with others undergoing the same experience. Language and memory barriers were easier to overcome when practising within a group of peers. Some of the participants explained that they did not feel judged or misinterpreted in this type of setting, and they felt comfortable practising:

*‘It means a lot, because you are allowed to speak without the comments of ‘what is he talking about?’ or an obvious shutdown in communication—because these people are in the same situation. It is good training, and good rehabilitation.’* (case 2)

In the Central Denmark Region, most of the participants appreciated the group training or special conversation groups with peers throughout the one-year process. In North Norway, none of the participants had stories that included peers while being followed up in the municipalities. The narratives indicated that peers were important for recovery, as well as established, continuous friendships across age groups and genders outside of organized group activities. Peers were referred to as a community of “us”, and they shared a collective *belonging* that helped them make sense of the world due to their common experiences. We found that belonging through enriching social relations had a positive influence on reconstruction of the embodied self and QOL.

### Becoming through follow-up and continuity in professional support

The initial recovery process during hospitalization and subacute rehabilitation was portrayed as a mainly positive experience for the participants. In this period, they typically experienced rapid progress in their function, and several of the participants highlighted the value of intensity in training, knowledgeable health personnel and good relations with peers and professionals.

When returning to their homes and undergoing follow-ups in the municipalities, a discrepancy was noted in how the participants described the continuity and follow-up by health professionals and how this affected their process of recovery and QOL. Participants from Denmark indicated that they received good care within the system, while most participants from Norway described discontinuity and insufficient follow-up. The participants reported that structural and organizational differences between health-care systems had an impact on QOL in several ways. For most of the participants, a close, continual follow-up was important for function, independence and return to “familiarity” and QOL, while for others, it was important for feeling safe and taken care of within the system.

In the Central Denmark Region, most of the participants described relatively continuous transitions between the health-care levels, and a system that worked optimally with very good support and help every step throughout the journey. Other participants from Denmark noted that the long-term continuity in follow-up services had provided them a smooth transition back to “normality” and the familiar self, influencing their experience of QOL.

‘It is the process in itself, the long-term plan that was made for me in the beginning, that has brought me back on my feet again. My life has become reasonable again.’ (case 2)

Most participants from North Norway described both an absent and incoherent follow-up by professionals in the communities. Transition periods between health-care levels were especially vulnerable; they did not describe seamless shifts, but rather disturbing breaks in transitions that affected their QOL. Most of the descriptions were related to the shifts between specialist health services (i.e., hospitals) and follow-up services in the municipalities. Another topic was vulnerability in the small municipalities related to professional follow-ups during holidays and sick-leaves, which could result in weeks or months without any training. Specificity in training was another concern. Thus, the participants from North Norway requested both quantity and quality in their follow-ups. One Norwegian participant related the lack of intensity and the discontinuity in professional support directly to his slow physical recovery and QOL. Months after his initial hospitalization, he was granted a stay in a rehabilitation institution that allowed an important transformation in his recovery:

*‘Of course, it would have helped me* [more treatments in the municipality]. *I saw what a three-week stay in a rehabilitation institution did for me. In 14 days, I doubled the strength in my hand.’* (case 11)

The participants related professional support to how they managed one year following stroke and their overall QOL. Participants from North Norway described a lack of municipal follow-up regarding several aspects to facilitate recovery towards a new everyday life. Another consequence was that the Norwegian participants did not portray a holistic recovery that matched that of the Danish participants. We found strong relationships between stories of person-centred approaches, continuity of support and reconstruction of the embodied self. The substance and continuity of professional support may therefore be of great importance to stroke survivors’ *becoming* by facilitating an individualized and tailored practice that supports reconstruction of the embodied self, hence accelerates the experience of QOL.

## Discussion

### How stroke challenges the embodied self and being in the world

The unfolding phenomena of QOL during the first year following stroke in North Norway and in the Central Denmark Region can be described by three different, entangled, embodied processes of reconstructing the embodied self. We identified these processes as *the familiar self, the unfamiliar self* and the *recovery of self*. We demonstrated that reconstruction of the embodied self was related to progress in functional recovery or adjustments for engaging in meaningful activities in life, which was important for QOL.

Previous studies have highlighted QOL as a negotiation of self and identity (Clarke & Black, 2005; Moeller & Carpenter, 2013). However, the findings of our study illustrate the interrelations between body, self and QOL in the unfolding recovery process after stroke. Embodied self in this study is understood as an interwoven relationship of body and self, where embodiment plays a central role in structuring experience, cognition and action (Gallagher, 2011). Our findings indicate that bodily changes influence an individual’s embodied self as others have also suggested (Kitzmüller et al., 2013; Pallesen, 2014; Timothy, Graham, & Levack, 2016). Studies have described recovery after stroke as a negotiation trajectory between body, self and participation in everyday life (Arntzen et al., 2015; Arntzen, Hamran, & Borg, 2015). Yoshida (1993) previously described the impact of chronic illness on self among adults with traumatic spinal cord injury. As in this study, the familiar self (conceptualized as the former self) refers to the pre-injury embodied self-concepts of an individual, which forms the basis for reconstruction of the embodied self.

### Quality of life—an integrated process of being, doing, belonging and becoming

We demonstrated that the negotiating processes of embodied selves were closely related to the individuals being, doing, belonging and becoming: Possibilities, relationships and support were significant for how the embodied self and QOL were perceived. The experienced differentiations and contextualization in reconstructing an embodied self after stroke are described less extensively in the literature, although Timothy et al. (2016) described a fluctuation of divergence and cohesion in the relationship between body and self following stroke.

### Being

Although fatigue is common immediately after stroke, tends to persist and contribute to lower QOL (Wu, Mead, Macleod, & Chalder, 2015), only the Danish participants described how professional support made their *being* easier through structured plans and coping strategies for managing on a daily basis. Previous authors (Meijering et al., 2016) have implied that stroke rehabilitation services should address the individual and everyday challenges to improve well-being. More research to understand destructive post-stroke phenomena, such as fatigue, has been requested to identify effective methods to help stroke survivors gain wholeness of body and self (Kitzmüller et al., 2013). Our findings indicate that professional support aimed to render different activities less time-consuming and demanding to prevent tiredness from taking up too much of the individual’s total capacity in everyday life, may support reconstruction of the embodied self and QOL through a more manageable *being*.

Stroke challenged several of the participants’ understanding of themselves in relation to *being* through disruption of, e.g., social roles (*being* a mother, *being* a co-worker), or cultural Western aspects of *being* with individual choice and agency. *Being* is associated with the lived experience and with the embodied structures that are used to make sense of the world and one’s meaningful activities, interactions or goals and to protect one’s sense of basic worth (Gallagher, 2011; Leary, Tangney, & ProQuest, 2012; Wilcock, 2006). Thus, the embodied self is related to existential aspects of human *being* that acknowledge the lived experience of “who I used to be”. Through the concept of *being*, fatigue can be understood as a disruption between the familiar and unfamiliar selves. Studies have highlighted the importance of intervening against fatigue (McGeough et al., 2009; Vestling, Ramel, & Iwarsson, 2013). The findings of this study illustrate the importance of reassuring how stroke survivors manage in life over time and the significance of professional support in the structure and manageability of everyday life to enhance QOL as much as possible.

### Doing and meaning-making

*Being* is linked to *doing* by action or engagement (Hitch et al., 2014a; Lyons, Orozovic, Davis, & Newman, 2002). We showed that certain elements, e.g., work, were more important than others for the embodied self and accordingly for QOL. Returning to work has been found to be an important factor for subjective well-being and satisfaction following stroke (Vestling, Ramel, & Iwarsson, 2005; Vestling, Tufvesson, & Iwarsson, 2003). Proportionally, more time is spent on work rather than other activities (Vestling et al., 2013), and in various ways, work was an integral part of identity and “who I am” and “what I do” for several of the participants, as described by others (Brannigan et al., 2017; Kielhofner et al., 1999). Although many of the participants were content with other or different work tasks, others were very expressive in saying “this is not me”. This finding emphasizes not only the importance of returning to work after stroke but also returning to previous work assignments (or other valued activities) related to self-worth and identity if this is significant to the individual. Previous studies indicated that the more a working role defines a person’s identity, the more essential work is to that person (Kielhofner et al., 1999). As portrayed by Vestling et al. (2013), work has multiple subjective meanings, where self-worth and social aspects are prioritized above the economic perspective of working. For most of the participants, work was regarded as enjoyable, enriching and meaningful. Productivity, personal development and performance are aspects relevant to the self in a work setting (Brannigan et al., 2017). As addressed in our study, sustained social relationships and collective meaning-making with co-workers was another important perspective, which has also been described previously (Brannigan et al., 2017; Vestling et al., 2013).

### Belonging and shared meaning

*Belonging* is identified with interpersonal relationships, connectedness and health (Wilcock, 2006). Enriching social relations were important for the participants’ QOL. This finding has been highlighted by others, e.g., Lynch et al. (2008), who stated that maintenance of healthy social relationships may be the most important and salient influential factor on QOL after stroke. King (1996) found that social support was essential to post-stroke QOL. Our findings with many and varied narratives of support, help, strengthened relationships, motivation, unity and shared meanings in these connections, suggest that both social interactions and continual close relationships are relevant for reconstruction of the embodied self and QOL. The aspect of *belonging* is multifaceted, and an individual may *belong* to multiple, different social networks, e.g., close family and friends and more formal settings such as co-workers or groups with peers. The connectedness through shared meaning that many participants had with peers was prominent and important for reconstruction of the embodied self. This finding suggests the value of more extensive establishment of groups of peers, especially in North Norway where none of the participants had experiences of such arrangements in the municipalities.

### Becoming through change and development

Our study illustrated that restoration of function and managing through functional progress or adjustments following stroke had existential value and was important for QOL. Previous studies disagree on the relationship between function and QOL (Carod-Artal & Egido, 2009; Clarke & Black, 2005; Samsa & Matchar, 2004). Our study demonstrated that the body’s ability to perceive and experience the world was abruptly disrupted, creating an ambivalent relationship between the familiar and unfamiliar embodied selves. The body is anchored in familiarity (normality), thus can create new familiarity through recovery. Further, functional recovery was important for all aspects of being, doing, belonging and becoming, and all dimensions were interconnected to the embodied self and the existential *being*. For these participants, the sense of *being* was built through a sense of *doing* meaningful things. Being able to do things evolved the sense of *becoming* and sustained the sense of *belonging*, emphasizing the connectedness between function, embodied self and QOL.

The dimension of *becoming* is related to a perceptual process of change and development that depends on stimulation or feedback from others, which is captured as a “situatedness” within ongoing life (Hitch et al., 2014a; Wilcock, 2006). *Becoming* is related to a dynamic and emergent perspective on identity, which is embodied by the changing self (Wilcock, 2006). Following stroke, professional support and therapeutic relationships essentially influence the development of *becoming* in the recovery process. Becoming is not always about improvement but also about adjustment through managing and maintaining (Hitch et al., 2014a). Our study found that professional support was crucial for change and development following stroke; thus, a difference was found between the countries in continuity and sustained support by professionals, which has also been described by Arntzen, Moe, Aadal, and Pallesen (2019), Pallesen, Aadal, Moe, and Arntzen (2019) and Aadal, Pallesen, Arntzen, and Moe (2018). Especially in Denmark, disabilities were continuously challenged through functional restorations or adjustments that positively impacted reconstruction of the embodied self among the participants. “Having a go” and “learning new things” are related to *becoming* (Lyons et al., 2002) and support the meaning and importance of professional follow-ups that aim to facilitate change and challenge development by optimizing functions throughout recovery, thus supporting reconstruction of the embodied self and QOL.

### Study strengths and limitations

The data material was rich and generated by experienced interviewers. Several interesting themes emerged that are not presented in this article. However, the presented analytic text is the most salient in terms of the content and meaning that emerged through the empirical data from the perspectives of a research team with various experiences and knowledge bases. The research team’s close knowledge of the data material strengthens the trustworthiness of this study. Reflexivity was central to the interpretative process, and the collective viewing, systematic analysis process and discussions among the authors challenged assumptions across an interdisciplinary research team (3 PTs, 2 OTs, 1 nurse and 1 MD). The findings in this study can be viewed as the best understanding that we have been able to develop and not a statement of the ultimate reality.

To assure that the aim of the study was emphasized, a semi-structured interview guide was collectively developed and used. Further, the interviewers encouraged the informants to talk about their experiences and perspectives by posing open questions. The participants were challenged to lead the conversation, encouraging the interview process to remain as close to the lived experience as possible. Since this study is part of a larger study, the interviewers and participants had previously met through earlier interviews. These established relationships may be a strength of the study by encouraging safety, trust and openness in the conversations. A few conversations involved participants’ close family members or others contributing to the study, which may have affected the dynamic between the involved participants and how life or recovery was portrayed. However, in our experience, their contributions were kept to a minimum, and their statements usually elaborated or clarified the meaning of the participants’ commentary.

A potential limitation may be that the group of participants included young stroke survivors. Reasonably, certain elements of important aspects for QOL would be different for younger individuals compared to a more average-aged group of stroke survivors. Nevertheless, the findings of this study elaborate on themes that may be helpful for clinicians working with younger stroke survivors. Stroke survivors constitute a heterogenous group, implying that interviews with other participants may have provided other perspectives regarding our research questions, as well as other possible perspectives within and between the two country regions. However, the abstraction process during the analysis supports the findings in this study as significant beyond the individual context.

## Conclusion

This study demonstrates different aspects of the embodied self and variations of reconstructing the embodied self one year following stroke. Although the recovery processes and contexts were different, the self-reconstruction process emerged as important for QOL in both countries. We identified three intertwined and negotiating processes: a familiar self, an unfamiliar self, and a recovery of self. Reconstruction of the embodied self was interconnected to bodily changes and functions. Enriching social relations, resumption of activities, successful return to work, and continuity and presence in professional support during the recovery process positively influenced QOL. The described fatigue and reported sustained reduced function, influenced the perceived QOL negatively. The variances in professional support revealed differences in continuity and sustained support between the Central Denmark Region and North Norway and how such differences affected reconstruction of the embodied self.

## Clinical implications

In a profound manner, QOL is related to the existential embodied self; how we see ourselves and our possibilities for meaningful interaction with our surroundings. Reconstruction of the embodied self is a means of understanding stroke survivors in the recovery process and has clinical value throughout the various stages of stroke rehabilitation. The intertwined and different aspects of the embodied self inherent to the individual stroke survivor are useful for supporting the evolving self towards a known and familiar self following stroke. Professionals may support the *being, doing* and *becoming* aspects of reconstruction of the embodied self by optimizing restoration of functions, facilitating the development of coping strategies and supporting adjustments in everyday life. Further, professionals can facilitate the *belonging* aspect, e.g., by establishing groups of peers and striving for return to social activities that are important to the individual. The findings indicate the value of continuity in services to support reconstruction of embodied self and QOL among stroke survivors. The complexity and individuality in reconstructing the embodied self are relevant for a personalized and tailored practice aiming towards important and meaningful aspects and activities for the individual—consequently improving their experience of QOL.
